# Supplementary material for: Complexity and Entropy in Physiological Signals (CEPS): Resonance Breathing Rate Assessed Using Measures of Fractal Dimension, Heart Rate Asymmetry and Permutation Entropy
Source: Entropy (Basel). 2023 Feb 6;25(2):301. doi: 10.3390/e25020301 (PMC9955651; doi:10.3390/e25020301)
Supplement: Supplementary file 1 [file entropy-25-00301-s001.zip › entropy-2104737-supplementary.pdf]

Supplementary Materials

This Supplement contains subsidiary information that was considered might detract from the presentation of the main arguments in the paper itself. It contains five Sections:

SM1. Parameter tuning

SM1.1. Parameter tuning – some results

SM1.2. Parameter tuning – plots of selected measures against parameters (11 Plots, A-K, in 35 parts)

SM2. Changes in CEPS measures over time

SM2.1. RRi data, resampled at 4 Hz

SM2.2. EDA data

SM3. Effects of age, sex, perceived stress and other trait and state measures on CEPS and Kubios HRV measures

SM4. Respiration and Asymmetry

SM4.1. Effects on outbreath-to-inbreath Respiration Ratio (RespR) and Rate

SM5. Some findings on correlation

SM5.1. Correlations within 'families' of measures, and between individual measures

SM5.2. Correlations within 'families' of measures, and between individual measures when applied to different data types (RRi, respiration and EDA)

SM5.2.1. Correlations between fractal dimensions within the same dataset

SM5.2.2. Correlations between datasets for individual fractal dimension measures

SM5.2.3. Correlations between HRA indices within the same dataset

SM5.2.4. Correlations between datasets for individual HRA indices

SM5.2.5. Correlations between the PE-based measures within the same RRi or respiration interval dataset

SM5.2.6. Correlations between data types for eight individual PE-based measures

SM1. Parameter tuning

SM1.1. Parameter tuning – some results

Many measures implemented in CEPS require the setting of one or more parameters before they can be calculated. To maximise measure values, or differences between their values in different states, often necessitates 'parameter tuning' appropriate to particular research objectives, as described in the main text of this paper. Note that different parameters may well be needed for different datasets and objectives. Optimally, all *n* parameters for a given measure should be tuned simultaneously in *n*-dimensional space in order to avoid order effects, but given the current limitations of CEPS, here they were tuned sequentially. For the first parameter tested, the others were set at values that seemed appropriate from the literature.

Results of parameter tuning to maximise the difference between Baseline and resonance breathing (RBR) trials for the non-resampled ECG RR interval data in this study ('RRi (noR)') are presented in Table SM1 below. This is followed by a selection of plots that show how results may vary as parameters are changed, or with the objective of maximising differences between Baseline and breathing at 5 BrPM (breaths per minute) rather than between Baseline and RBR.

Table S1. CEPS measures, in alphabetical order, with parameters tuned for each measure, listed in the order in which they were tuned. Numbers in parentheses indicate ranges tested, based in part on indications from the literature. For the abbreviations used, see the main paper. Abbreviations not listed there are explained below. In this and the following Tables, alternating rows have been given a coloured background simply to aid readability.

Measure	Parameter 1	Parameter 2	Parameter 3	Parameter 4
AAPE	Order 8 (2-8)[a]	*tau* 3 (1-4)	*A* 0.5 (0.1-1.0)	n/a
ACR	Lag 5 (1-30)	n/a	n/a	n/a
ACV	Lag 1 (1-30)	n/a	n/a	n/a
AE	Scale 30 (1-30)	Bin size 30 (5-100)	n/a	n/a
AF	*n* wind 30 (1-30)	n/a	n/a	n/a
AMI	*tau* 18 (1-20)	n/a	n/a	n/a
AttnEn	Scale 15 (1-20)	Log base[b]	n/a	n/a
AvgApEnP	*m* 1 (1-10)	n/a	n/a	n/a
AvgSampEnP	*m* 1 (1-15)	n/a	n/a	n/a
BE	*m* 15 (1-20)	n/a	n/a	n/a
CAFE	Type CenInv (1-8)	*m* 2 (2-20)	*r* 0.02 (0.02-0.40)	Mf 5 (1-10)
CCM	*tau* 1 (1-20)	n/a	n/a	n/a
CoSiEn	*m* 4 (2-10)	*tau* 1 (1-20)	r 0.10 (0.01-0.20)	n/a
CPEI	epz 0.06 (0.002-0.06)[c]	n/a	n/a	n/a
Disten	*m* 1 (1-20)	Bin *M* 8 (8-128)	*tau* 2 (1-20)[d]	n/a
EoD	*m* 10 (1-30)	*s* 1 (1-20)	n/a	n/a
EoE	Scale 10 (1-30)	Bin size 30 (5-100)	n/a	n/a
EPE	*m* 6 (2-8)	*tau* 1 (1-20)	*r* 0.2 (0.2-4.0)	n/a
EPP r	*k* 7 (1-20)	n/a	n/a	n/a
EPP SD1	*k* 7 (1-20)	n/a	n/a	n/a
EPP SD2	*k* 7 (1-20)[d]	n/a	n/a	n/a
ESCHA_c	minV 1 (1-4)	maxV 10 (2-10)	Dist 2 (2-20)[e]	n/a

FD_H	k_{max} 6 (2=15)	n/a	n/a	n/a
FE	mf *Z* local (5 methods)	*m* 2 (1-20)	*tau* 1 (1-10)	*r* 0.01 (0.01-0.30)
GridEn	*m* 2 (1-20)	*tau* 1 (1-20)	n/a	n/a
HRA GI	*tau* 1 (1-20)	n/a	n/a	n/a
HRA PI	*tau* 1 (1-20)	n/a	n/a	n/a
HRA SI	*tau* 2 (1-20)	n/a	n/a	n/a
ImPE	*m* 6 (2-6)	*tau* 1 (1-20)	Scale 1 (1-20)	n/a
IncrEn	*m* 3 (2-20)	*tau* 1 (1-20)	Log 1.5 (1.5-20)	n/a
KLD	*m* 10 (1-10)	*s* 1 (1-20)	n/a	n/a
LLE	max iter 44 (1-50)	*MP* 2.0 (0.5-5.0)	*tau* 27 (1-30)	*m* 1 (1-30)
mFD_M	*n* wind 5 (1-30)	max scale 5 (2-30)	n/a	n/a
mLZC	Scal 15 (1-31)	n/a	n/a	n/a
MmSE	*m* 15 (1-20)[d]	n/a	n/a	n/a
mPE	scale 1 (1-30)	*tau* 1 (1-10)	*m* 4 (1-4)	n/a
mPhEn	*k* 2 (1-30)[f]	scale 2 (1-20)	n/a	n/a
mPM_E	*m* 5 (2-10)	*tau* 1 (1-10)	n/a	n/a
NLDiL_m	wind 10 (1-30)[d]	Step 10 (1-10)[d]	n/a	n/a
NLDiP_m	wind 10 (1-30)[d]	Step 10 (1-10)[d]	n/a	n/a
NLDwL_m	wind 10 (1-30)[d]	Step 10 (1-10)[d]	n/a	n/a
NLDwP_m	wind 10 (1-30)[d]	Step 10 (1-10)[d]	n/a	n/a
PhEn	*k* 2 (1-30)	n/a	n/a	n/a
PJSC	*m* 5 (1-10)	*tau* 2 (1-20)	n/a	n/a
RCmDE	*m* 3 (2-4)	*c* 3 (1-10)	*tau* 3 (1-20)	Max scale 12 (1-20)
RE	*q* 0.5 (0.05-0.5)	n/a	n/a	n/a
RPE	*m* 4 (1-10)	n/a	n/a	n/a
RQA DET	*m* 6[d]	Min line 2[d]	*tau* 3 (1-30)	*r* 2.4495[d]
RQA ENT	*m* 6[d]	Min line 2[d]	*tau* 22 (1-30)	*r* 2.4495[d]
RQA LAM	*m* 6[d]	Min line 2[d]	*tau* 15 (1-30)	*r* 2.4495[d]
RQA Lmax	*m* 10[d]	Min line 2[d]	*tau* 9 (1-30)	*r* 2.4495[d]
RQA REC	*m* 6 (1-20)[d]	Min line 2[d]	*tau* 12 (1-30)	*r* 2.4495[d]
RQA TT	*m* 6 (1-20)[d]	Min line 2[d]	*tau* 10 (1-30)[d]	*r* 2.4495[d]
SEx	*m* 3 (1-20)	Num int 2 (1-20)	n/a	n/a
ShannonEntropy	bin 10 (1-10)	*m* 4 (1-10)	n/a	n/a
TE	*q* 0.05 (0.05-0.5)	n/a	n/a	n/a
T_E Entropy	lag 7 (1-30)	n/a	n/a	n/a
T_E Tone	lag 7 (1-30)	n/a	n/a	n/a
TPE	*m* 4 (1-10)	*tau* 1 (1-10)	*q* 1.1 (0.1-5.0)	n/a

a. AAPE is demanding to compute, so Order was only tested from 2 to 8; b. Not tested; c. The same result was obtained, whether or not the IQR method was used, suggesting a coding error; d. Value was chosen as a compromise; e. Test results would not save to Excel, so used default value; f. k = 2 was adopted from testing PhEn.

Abbreviations not explained in the main text:

AMI Auto-Mutual Information

ACV Autocovariance

EoE Entropy of entropy

FE Fuzzy entropy.

SM1.2. Plots of selected measures

These plots have been selected for their visual interest, and to demonstrate the usefulness of testing parameters in CE, not necessarily for their relevance to the results of our study on resonant breathing rate.

Three types of plots were created:

(1) Medians 1. Differences ("Diff") between group median values of a measure in two different trials (e.g., Baseline and RBR), or medians of the individual differences in measure values for the two trials ("mdn"), against the parameter of interest;

(2) Medians 2. Group median values of the measure against the parameter of interest, for the different trials;

(3) Counts. Where appropriate, numbers of positive and negative differences between the two trials, against the parameter of interest. For our sample of 44 participants, counts furthest from 22 (i.e., 44/2) would indicate that such differences are significant.

A. Kullbach-Leibler Divergence (KLD)

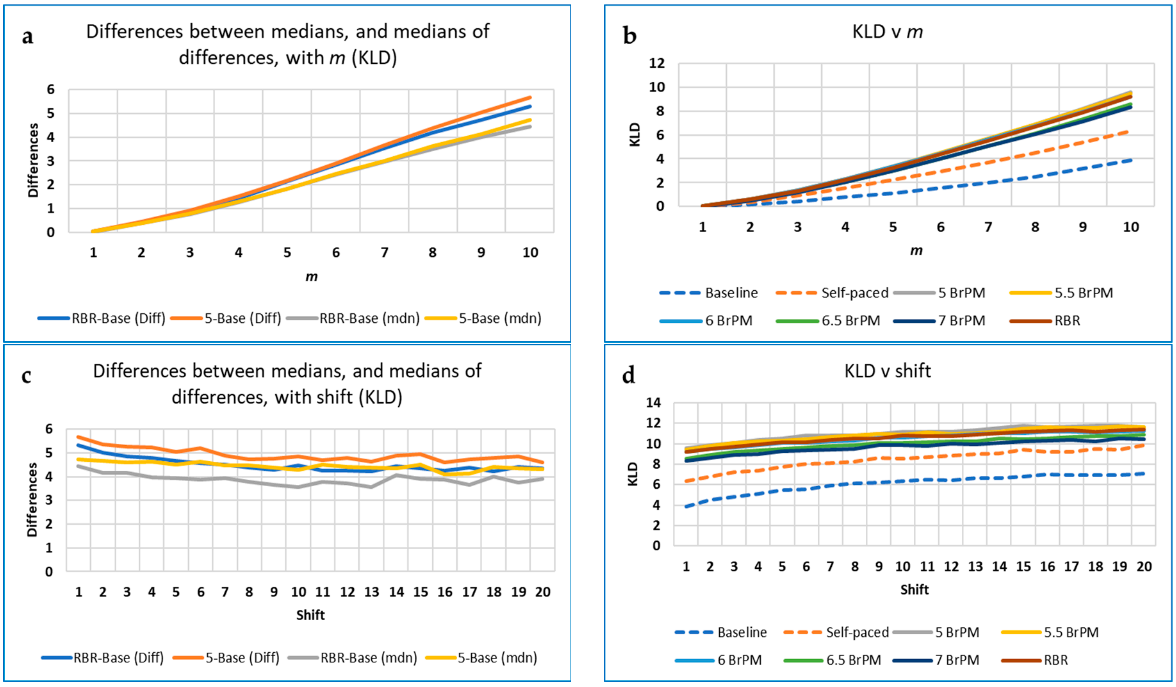

Values of KLD increase with *m*, but less so with 'shift' *s*. Difference between Baseline, Self-paced and the externally paced breathing rates are marked in the Type (2) plots (Right).

B. Increment entropy (IncrEn)

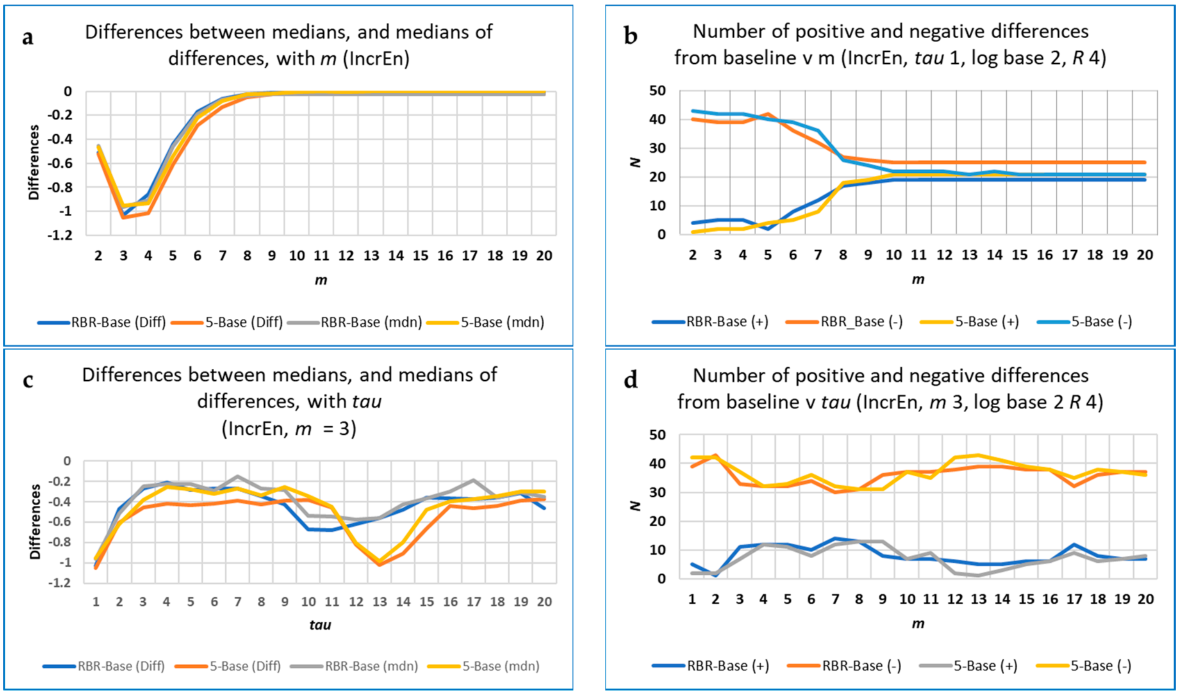

The Type (1) plots (Left) show marked minima for particular values of *m* and *tau*. These are reflected in the Type (3) plots (Right), where the counts are most different from 22.

C. Higuchi fractal dimension (FD_H)

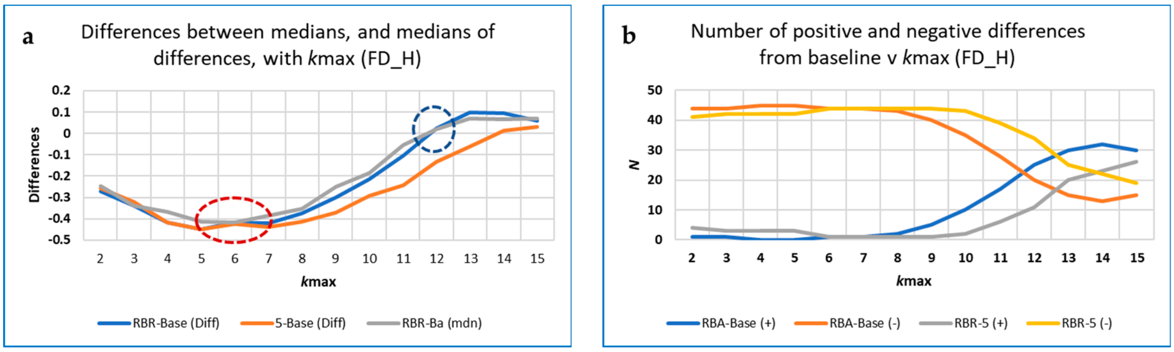

The Type (1) plot (Left) shows greatest difference from 0 at around $k_{max} = 6$, and least at $k_{max} = 12$. At both these values of k_{max}, the differences between the group median values of FD_H at Baseline and RBR converge with the corresponding medians of the individual differences in FD_H. The Type (3) plot (Right) confirms that greatest Baseline-to-RBR differences occur at around $k_{max} = 6$, with fewest at $k_{max} = 12$.

D. Largest Lyapunov exponents (LLE)

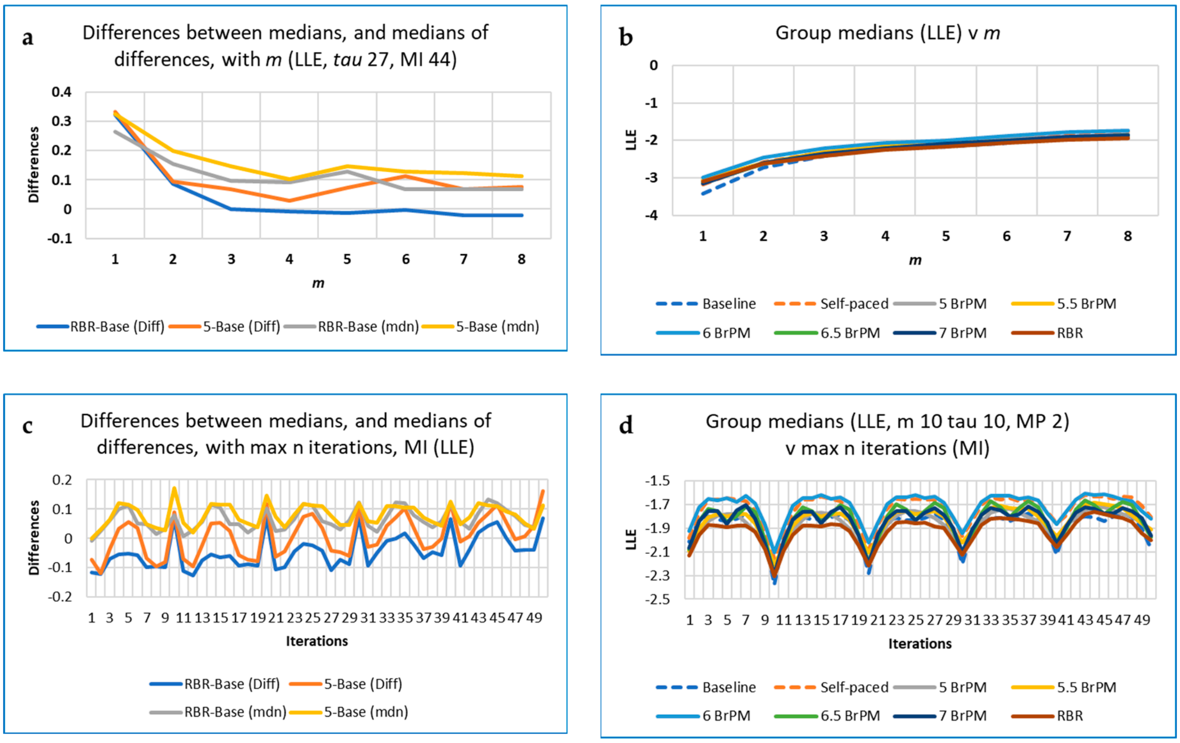

The Type (1) and (2) plots (Above) show Baseline-to-RBR and Baseline-to-5 BrPM differences decreasing with *m* (Left), while the actual LLE values increase asymptotically with *m*. The corresponding plots for differences and values against the maximum number of iterations (Below), are strikingly different.

Repeating patterns of changes with increasing parameter values are observed with other measures as well:

E. Autocorrelation (ACR)

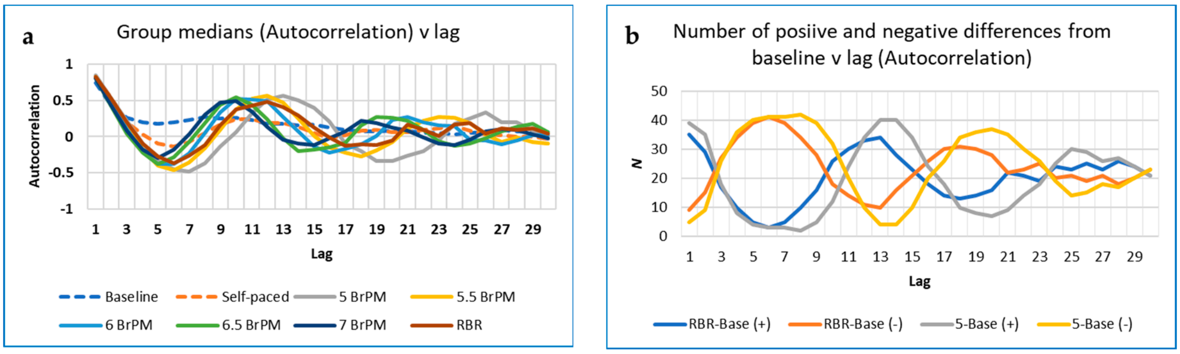

Both the Type (2) and Type (3) plots (and, indeed, the Type (1) plot, not shown here), demonstrate marked rhythmicity – least at Baseline, followed by Self-paced breathing, and particularly marked during externally paced breathing.

F. Extended Poincaré plot (EPP) – SD1, SD2 and *r*

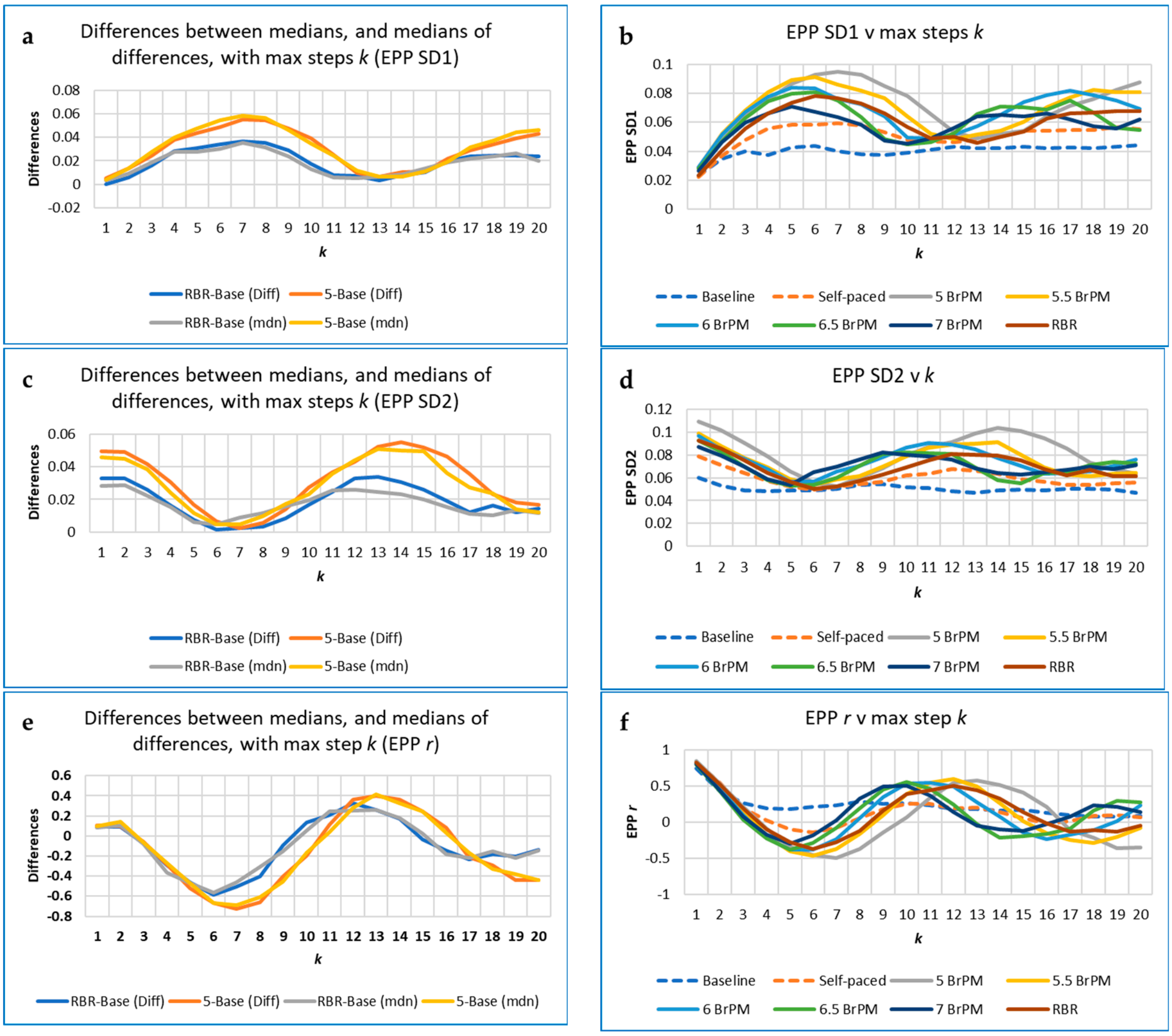

(Note the phase difference between SD1 and SD2.)

Wavelike patterns are also visible here, particularly for EPP *r*:

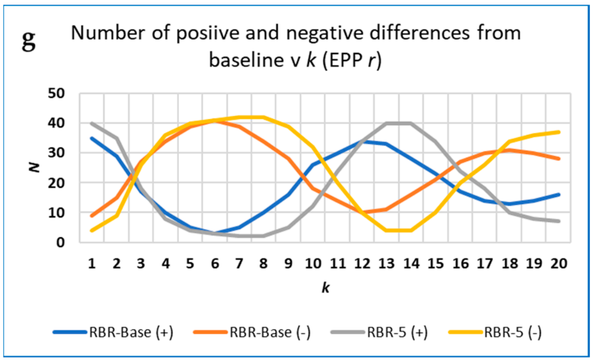

G. Tone-entropy (T_E) Tone

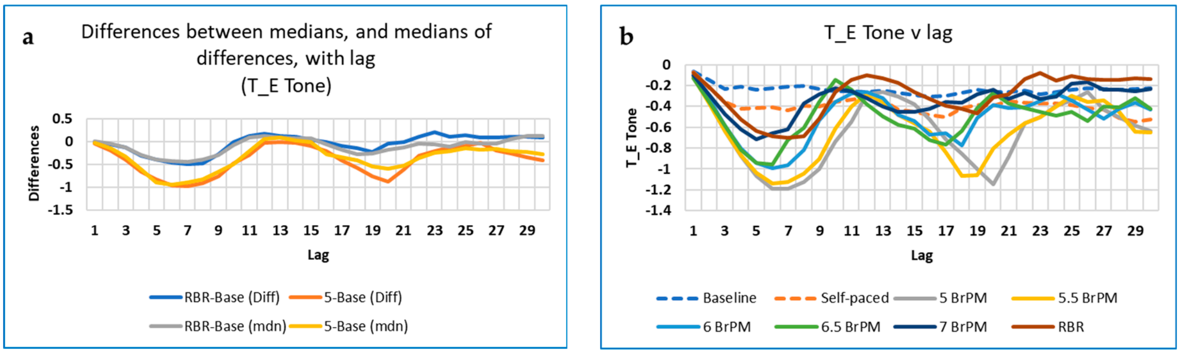

In some ways, these plots are quite similar to those for EPP SD2. As with EPP SD1 and SD2, there is a similar phase difference here (not illustrated) between T_E Tone and T_E Ent.

H. Gridded Distribution entropy (GridEn)

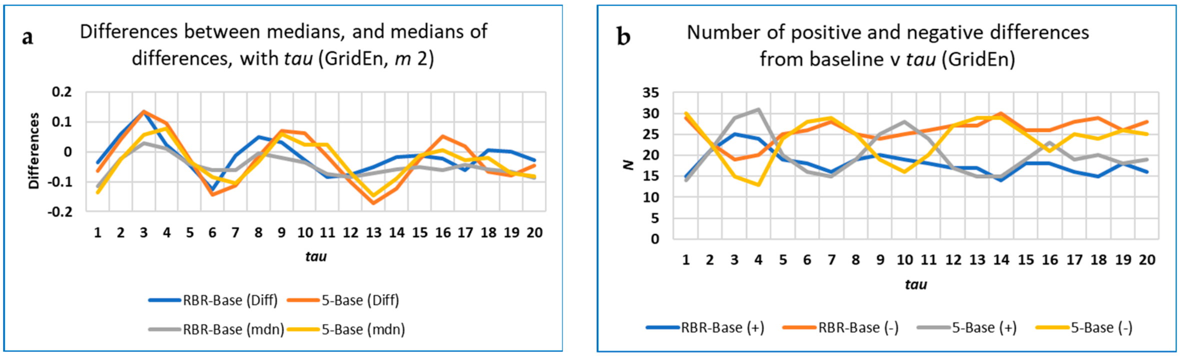

GridEn is based on a coarse-grained version of the Poincaré plot, so once again we see a rhythmical pattern with *tau*. However, here the maxima and minima occur at different lags than those for the EPP shown in F. Extended Poincaré plot.

I. Bubble entropy (BE)

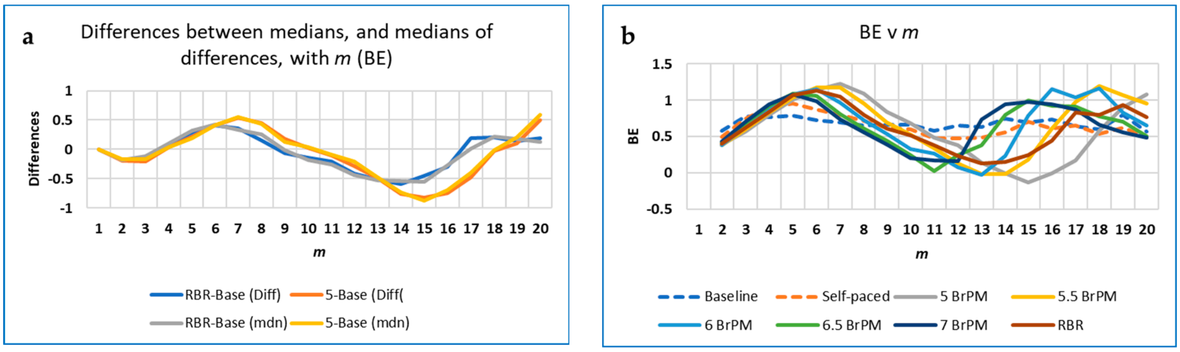

However, wave-like patterns are not solely the prerogative of measures derived from Poincaré plots. They also occur for BE, and here it is interesting to observe that while the traces for BE more or less coincide at around m = 5-7 for the different breathing rates, this is no longer the case for $m \approx$ 15-19.

J. Rényi permutation entropy (RPE) and Tsallis permutation entropy (TPE)

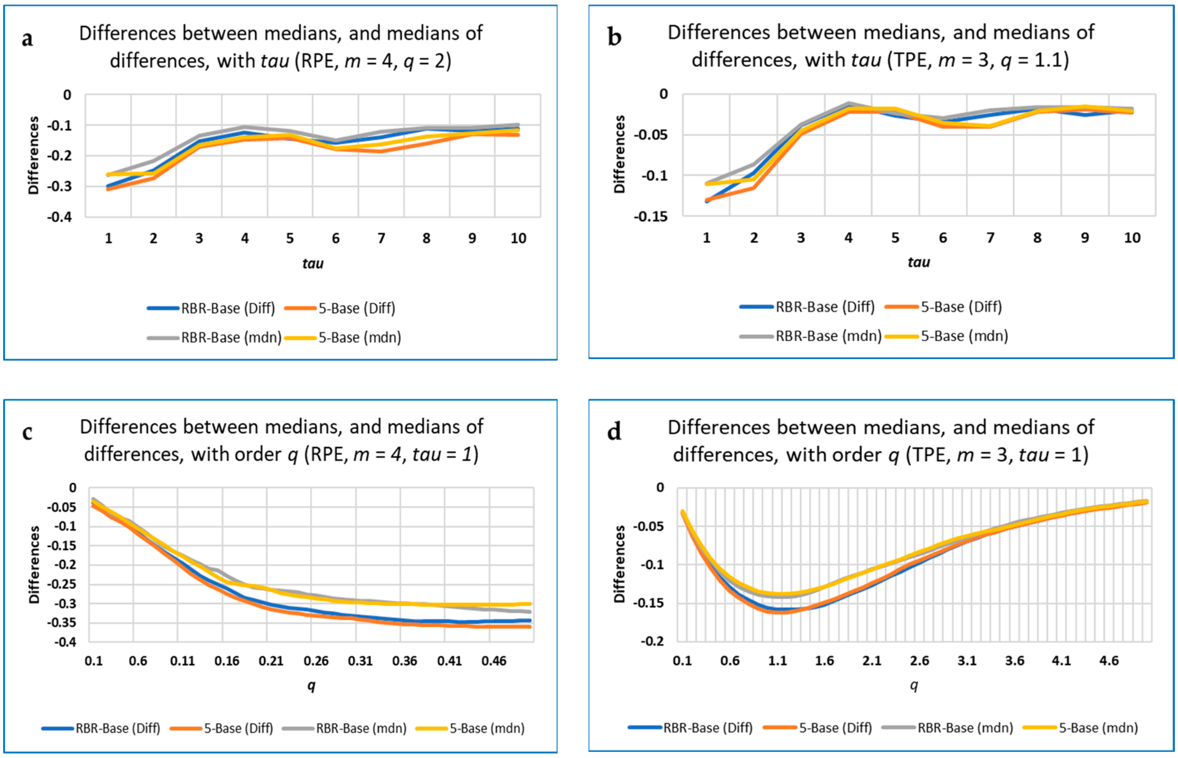

There is even something of a wave-like pattern when differences in both RPE and TPE are plotted against *tau* (a and b, above), although plotting such differences against order *q* results in very different graphs for the two measures (c and d, below).

K. Fuzzy entropy (FE), Z-shaped membership function (local)

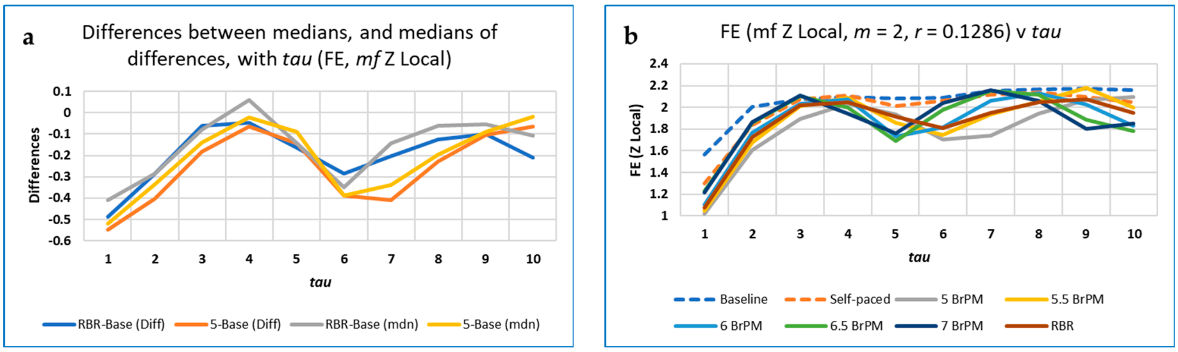

Again, a wave-like pattern is visible for this variant of FE.

SM2. Changes in CEPS measures over time

Changes over time were investigated for the RRi and EDA data (there were too few data points for this to be feasible for the respiration data).

SM2.1. RRi data, resampled at 4 Hz

With each 5-minute Trial divided into five 1-minute segments, most RRi FD, HRA and PE-type measures *increased* over time, whatever the breathing rate. These increases occurred in 35 or more of the 44 participants for FD_H, FD_P, FD_Dist, FD_Sign and mFD_M, for PI and $C2_a$, and for TPE, EPE and ImPE. Median FD values over the five minutes were lower at Baseline than during paced respiration for 18 of the 22 FD measures analysed, but higher at Baseline than during paced breathing for all 8 of the PE-based measures (13 of the 22 measures derived from Poincaré plots increased and nine decreased).

SM2.2. EDA data

With each 5-minute Trial divided into five 1-minute segments, the EDA entropy measures, especially the permutation entropies mPE1 and mPM_E, *decreased* over time, whatever the breathing rates, with values lower at Baseline than during paced respiration.

SM3. Effects of age, sex, perceived stress and other trait and state measures on CEPS and Kubios HRV measures

The effects of age, perceived stress, 'Mindful awareness' and the two dimensions of interoceptive awareness on how CEPS measures reflect breathing state were assessed by splitting the data into 'low' and 'high' parts with respect to median values. As no participants declared otherwise, sex was considered as a binary measure (Female/Male). Mann-Whitney tests were used to analyse whether the χ^2 and W values were significantly different for the two subgroups being compared. Correlations between physiological and questionnaire measures were also explored, as well as between the different questionnaire results – using Spearman's *rho* rather than Pearson's *r*, given that much of the data was not normally distributed.

Subgroup analyses were conducted using Mann-Whitney tests with a threshold of p = 0.01, so that only subgroup differences with a lower p-value were examined. The tests were carried out for measures at Baseline and during Self-paced breathing, and also for differences in measures between the Baseline or Self-paced trials and the composite RBR trials.

The following subgroups were considered: Female/Male, Older/Younger (45-84 vs. 18-44 years old), and – relative to the group median – high/low scores for the Perceived Stress Scale (PSS-10) and its Coping and Distress subscales, for the mean Mindful Attention Awareness Scale (MAAS) score, and for the Multidimensional Assessment of Interoceptive Awareness (MAIA) 'Noticing', 'Attention Regulation' and 'Self-Regulation' subscales.

Results were analysed for 219 RRi noR and RRi 4R measures, for 96 EDA measures (and an additional seven from DynamicalSystems.jl), for 102 measures from the 'raw' RSP data, for 200 RSP IN, 201 RSP OUT and 201 RSP PP measures, and for 60 Kubios HRV measures. This made a total of 1305 measures. As a very rough estimate, using the significance threshold of p = 0.01, around 13 (1%) of these measures might be expected to show Mann-Whitney test differences by chance alone.

Table SM2 shows that Age in particular affected many measures, although not for changes between Self-paced breathing and RBR. Sex, MAIA 'Noticing; and 'Attention Regulation' may also have affected some measures. On the other hand, 'Self-Regulation' did not appear to.

Table S2. Subgroup analysis, for all data types.

ALL	Baseline	Self-paced	RBR_Base	RBR_Self
Age	189	129	122	6

Sex	2	1	17	10
PSS-10 Total	2	1	10	4
PSS-10 Coping	4	2	4	1
PSS-10 Distress	4	1	1	4
MAAS mean	5	4	5	0
MAIA Noticing	2	16	7	5
MAIA AttnReg	0	5	0	15
MAIA SelfReg	3	13	3	4

When results were divided by data type *and* trial (or trial-to-trial change), only Age and MAIA 'Noticing' still showed more than 10 comparisons with p < 0.01, as shown in **Tables S3** and **S4**.

Table S3. Subgroup analysis for the effects of age on measures, for all data types (p < 0.01).

AGE	Baseline	Self-paced	RBR_Baseline	RBR_Self-paced
RRi (noR)	58	42	46	1
SRRi (4R)	105	66	60	2
EDA	1	1	1	0
Raw RSP	2	3	1	2
IN	2	4	2	0
OUT	0	0	0	0
PP	4	0	0	0
Kubios HRV	19	13	12	1
**ALL**	189	129	122	6

Table S4. Subgroup analysis for the effects of MAIA 'Noticing' on measures, for all data types (p < 0.01).

MAIA Noticing	Baseline	Self-paced	RBR_Baseline	RBR_Self-paced
RRi (noR)	0	0	3	0
SRRi (4R)	2	1	4	0
EDA	0	0	0	2
Raw RSP	0	**10**	0	1
IN	0	0	0	0
OUT	0	5	0	2
PP	0	0	0	0
Kubios HRV	0	0	0	0
**ALL**	2	16	7	5

MAIA 'Noticing' appears to have some effect on measures during Self-paced breathing, but not otherwise. Given that 'Noticing' covers 'awareness of body sensations', and that Self-paced breathing would require at least some such awareness, this might have been expected.

All ten Raw RSP measures for which p < 0.01 were *lower* in those participants with greater 'Noticing' scores (7 FDs, 3 PE-based measures). Measures that were higher in those with greater 'Noticing' scores were two 'other entropies' (SEx and GridEn), both for OUTbreath data.

From **Table S3**, it is clear that Age affects CEPS measures based on the RRi data (and thus the Kubios HRV measures as well) but has very little effect on measures from the EDA or RSP/breathing interval data.

Focusing only on those measures for which p < 0.01 for both the RRi (4R) *and* RRi (noR) data, the effects of age at Baseline on the RRi (4R) measures are shown in **Table S5**. Usually, the effects of age were similar at Baseline these and on how measures changed between Baseline and the composite RBR trial, but for some measures effects were opposite. As an example, in general FD values decreased with age at Baseline, as did the difference between their values at Baseline and during RBR, but this difference increased for FD_K.

Table S5. Effects of Age on CEPS measures for which p < 0.01 for both the RRi (4R) *and* RRi (noR) data at Baseline, showing opposite effects for difference between values of some measures at Baseline and during RBR.

**Family**	**Older > Younger**	Opposite for RBR-Baseline	**Younger > Older**	Opposite for RBR-Baseline
FD	0	0	8	1
HRA	0	0	2	2
PE-based	0	0	1	0
RQA	10	0	0	0
OC	3	0	4	4
OE	2	1	9	0
Other	2	0	6	4
**ALL**	17	1	30	11

For changes in values between Baseline and RBR, participant sex had an effect on seven RRi data CEPS measures, nine breathing interval CEPS measures (four linear, five 'Other complexities'), and one Kubios HRV measure. Focusing again on those measures for which p < 0.01 for both the RRi (4R) *and* RRi (noR) data, Sex had an effect only on Bubble entropy (BE). BE, together with all nine breathing interval measures, were greater for women than for men.

Mann-Whitney test results also suggested that differences in CEPS measures between Baseline and RBR trials might be greater in men than in women, and in poor than good copers (assessed using Cohen's Perceived Stress Scale, PSS-10). In other words, men might benefit more than women from paced breathing (as was the case for the standard HRV measures as well), and poor copers more than good copers.

Thus, for those who scored above the median on the perceived stress score (PSS-10), ten measures increased more between Baseline and the composite RBR trial than for those who scored below the median.

However, there were no significant differences in PSS (or PSS subscale) scores between men and women, or between older and younger participants (p > 0.5 for Sex, and p > 0.1 for Age, using Mann-Whitney tests), even though median PSS (and PSS Distress) scores were somewhat higher in men and older participants. Furthermore, no significant correlations were found between EDA CEPS measures and scores on Cohen's Perceived Stress Scale (PSS-10) or its stress and coping subscales.

On the other hand, the MAIA 'capacity to regulate attention' was found to correlate significantly with the total PSS-10 score (*rho* = -0.368, asymptotic p = 0.015), indicating that the higher the PSS, the lower the ability to regulate attention; there was also a negative correlation between PSS and Mean MAAS (*rho* = -0.448, p = 0.003), again suggesting that higher PSS might be associated with lower trait mindfulness. Incidentally, in this group, Mean MAAS also correlated positively with Age (*rho* = 0.350, p = 0.020).

SM4. Respiration and Asymmetry

For the 284 respiration recordings, the median Outbreath-to-Inbreath ratio (RespR) was 1.424 (IQR 1.239 to 1.650). For eight participants, 17 recordings resulted in three or more *negative* ratios, suggesting mislabelling of peaks and troughs by ProcessSignals. Fifteen of these were for Baseline or Self-paced breathing. It thus appears that ProcessSignals had more difficulty in recognising peaks and troughs for these than for the Paced breathing slots.

RespR for the group was more often less than the group median (1.424) at baseline, during self-paced breathing and for the RBR, but more often greater than the median for paced breathing rates > 5 BrPM, and equal to the median at 5 BrPM, the most common paced breathing rate (Figures SM1, SM2). Clearly, participants breathed in and out more equally during 'normal slow' or 'self-paced' breathing than during externally paced breathing with RespR deliberately ≠ 1. There was correspondingly less variance (RoCV) in RespR during the externally paced breathing trials.

Given this difference in 'respiration asymmetry', the HRA measures might be expected to correlate more strongly with RespR than some of the other families of measures, but in fact relatively fewer and weaker correlations with RespR occurred for the HRAs than for FDs and, particularly, than for the PE-based measures. For the latter, the absolute value of Spearman's *rho* was greater than 0.6 for 75% of all correlations with RespR at Baseline (although not for CPEI); *rho* was also > 0.6 for 50% of all correlations during self-paced breathing (including all three CPEI correlations – for noR, 4R and 10R data). In contrast, there were no correlations having *rho* > 0.6 during externally paced breathing. For the FDs, corresponding percentages were 27.3% (Baseline) 22.7% (Self-paced) and 0% (externally paced). In contrast, there were no correlations in any trial between HRA measures and RespR for which $|rho| > 0.6$.

For RespR itself, calculated using two different methods, Friedman's χ^2 was > 189.9, and Conover's *S* > 14.2. In other words, some of the derived CEPS measures were paradoxically more able to differentiate between breathing rates than RespR itself.

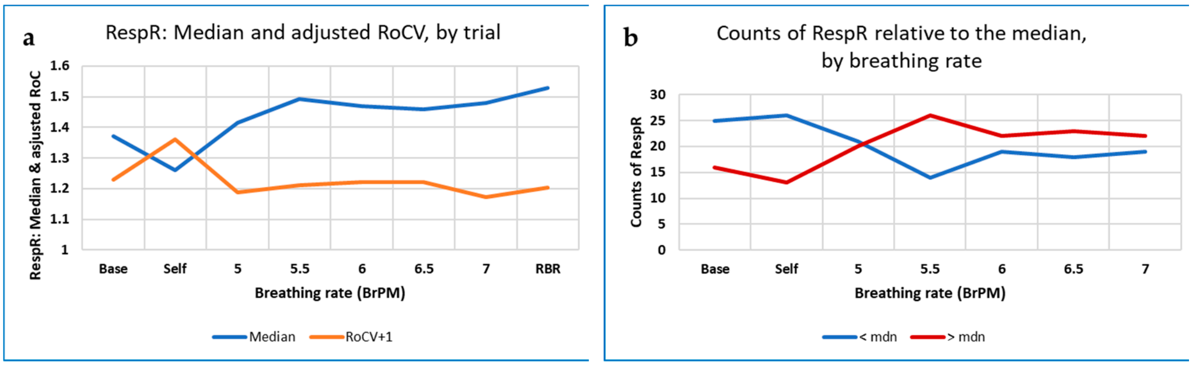

Figure S1: (a). Median OUTbreath-to-INbreath ratio (RespR) for the different trials, with its scale-adjusted Robust coefficient of variation (RoCV); (b) Counts of RespR relative to the median, by trial.

SM4.1. Effects on outbreath-to-inbreath Respiration Ratio (RespR) and Rate

Significant differences in respiration ratio (RespR) were found at Baseline between older and younger study participants, using the Mann-Whitney test (N = 40, Z = -2.066, ES = 0.327, p = 0.039). At Baseline, RespR was lower in older participants (median, IQR: 1.317, 1.104 to 1.414) than in younger participants (1.464, 1.207-1.619). This was also the case during Self-paced breathing (medians 1.239 vs. 1.308), but of the externally paced breathing trials only for pacing at 5.5 BrPM.

Results were more clear-cut for Sex, with RespR consistently higher in women than in men for all trials (Figure SM2(a)), particularly during Self-paced breathing.

During the paced respiration trials, median RespR was 1.55 for women, and only 1.41 for men, indicating that men may have found it more difficult to follow the onscreen pacer. Self-assessed pacing accuracy was in fact recorded on a 0-10 scale during the five paced Slots, and median accuracy was indeed marginally lower for men than for women (0.7, as against 0.8).

RespR were also consistently higher in those with lower PSS scores (Figure SM2(b)). Indeed, when breathing at the RBR, RespR was inversely correlated with both PSS score (*rho* = -0.379, p = 0.019, 2-tailed) and the PSS stress subscale score (*rho* = -0.339, p = 0.037).

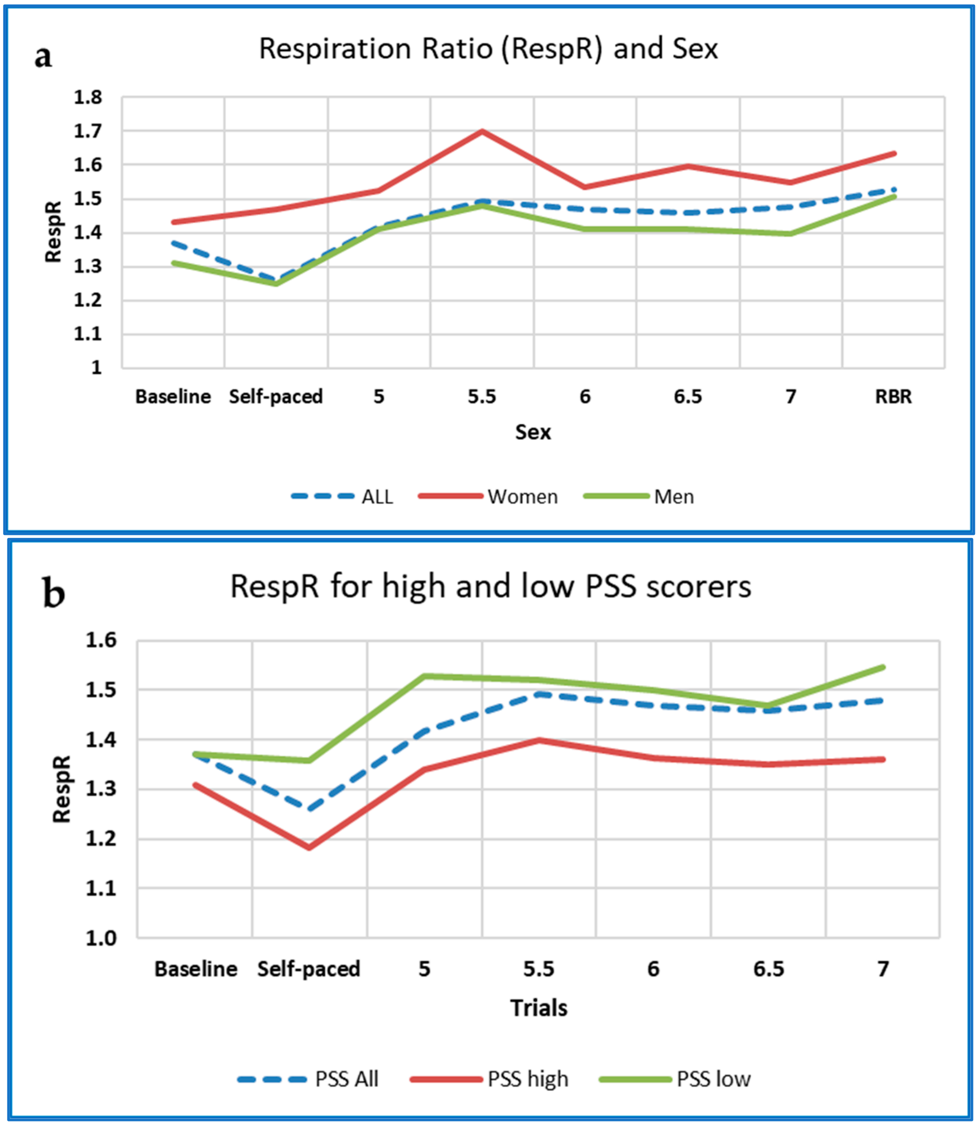

Figure S2: (a) Respiration ratio (RespR) and Sex; (b) RespR and PSS score.

Respiration *rate* (as opposed to ratio) was higher in those with greater PSS (and PSS Distress) scores in all trials except when breathing at 5.5 or 6.5 BrPM. These differences were significant when breathing at 6 BrPM (Mann-Whitney test, $N = 44$, $Z = -2.476$, ES = 0.373, $p = 0.013$). In contrast, respiration rate did not differ significantly between women and men in any trial.

For all paced breathing trials, median respiration rate was greater than the target breathing frequency by around 0.220 BrPM (median 0.220, IQR 0.216-0.223).

SM5. Some findings on correlation

SM5.1. Correlations within 'families' of measures, and between individual measures

Spearman's *rho* rather than Pearson's *r* was used to explore correlations within 'families' of measures, and between individual measures when applied to different data types (RRi, respiration and EDA). Those measures in the FD and HRA families are listed in Tables 1 and 2 in the main paper. The third family consisted of measures based on permutation entropy (PJSC, mPE, ImPE, EPE, mPM_E, RPE, TPE and CPEI).

Pairwise correlations between measures were examined: Baseline with RBR or 5 BrPM; Self-paced with RBR or 5 BrPM. Correlations in the same paired trials for which Spearman's *rho* > 0.7 for all four comparisons were tabulated (Tables SM6-SM10).

Table S6. RRi data (4R). Number of measures with *rho* > 0.7 and the descriptive statistics for these (Q1, median, Q3), followed by the values of the four measures with greatest maximum values (highest values in each row indicated by bold type).

4R	Self to RBR	Base to RBR	Self to 5 BrPM	Base to 5 BrPM
N measures	9	19	5	5
Median *rho*	0.831	0.841	0.901	**0.950**
Q3 *rho*	0.816	0.812	0.873	**0.945**
Q1 *rho*	0.791	0.808	**0.862**	0.859
**Top 4 measures**				
ESCHA_c	0.919	**0.950**	0.932	0.949
Q3	0.930	0.934	0.943	**0.945**
RE	0.918	0.946	0.944	**0.959**
TE	0.929	0.941	0.944	**0.950**

Table S7. RRi data (noR). Number of measures with *rho* > 0.7 and the descriptive statistics for these (Q1, median, Q3), followed by the values of the four measures with greatest maximum values (highest values in each row indicated by bold type).

noR	Self to RBR	Base to RBR	Self to 5 BrPM	Base to 5 BrPM
N measures	3	13	5	11

Median *rho*	0.780	0.799	0.854	**0.890**
Q3 *rho*	0.797	0.831	0.864	**0.938**
Q1 *rho*	0.772	0.789	**0.842**	0.835
**Top 4 measures**				
ESCHA_c	0.924	0.941	0.936	**0.953**
LZC	0.927	0.920	0.931	**0.942**
Q3	0.938	0.944	0.943	**0.954**
TE	0.923	0.930	0.938	**0.945**

Table S8. Raw Respiration data. Number of measures with *rho* > 0.7 and the descriptive statistics for these (Q1, median, Q3), followed by the values of the four measures with greatest maximum values (highest values in each row indicated by bold type).

RSP Dedup	Self to RBR	Base to RBR	Self to 5 BrPM	Base to 5 BrPM
N measures	8	5	18	7
Median *rho*	**0.563**	0.431	0.555	0.473
Q3 *rho*	0.586	0.528	**0.640**	0.592
Q1 *rho*	0.286	0.225	**0.389**	0.286
**Top 4 measures**				
Q1	**0.839**	0.823	0.838	0.826
Q3	0.834	0.838	**0.850**	0.843
RE	**0.845**	0.834	0.836	0.823
TE	**0.847**	0.836	0.835	0.822

Table S9. RSP interval data (IN, OUT and PP). Number of measures with *rho* > 0.7 and the descriptive statistics for these (Q1, median, Q3), followed by the values of the four measures with greatest maximum values (highest values in each row indicated by bold type).

RSP IN intervals	Self to RBR	Base to RBR	Self to 5 BrPM	Base to 5 BrPM
N measures	0	0	0	0
Median *rho*	**0.138**	0.121	0.100	0.076
Q3 *rho*	**0.270**	0.269	0.266	0.216
Q1 *rho*	**0.029**	-0.007	-0.121	-0.041
**Top 4 measures**				
GI	**0.681**	0.435	0.412	0.225

EI	**0.677**	0.385	0.384	0.247
Hjorth mobility	0.431	0.423	**0.552**	0.253
ACR1	0.380	0.401	**0.546**	0.260

RSP OUT intervals	Self to RBR	Base to RBR	Self to 5 BrPM	Base to 5 BrPM
N measures	0	0	0	0
Median *rho*	**0.111**	0.095	0.101	0.076
Q3 *rho*	0.246	0.192	**0.276**	0.160
Q1 *rho*	**0.015**	-0.020	-0.121	-0.026
**Top 4 measures**				
NLDiP_m	0.446	0.197	**0..566**	0.221
NLDiL_sd	**0.555**	0.081	0.439	0.022
Jitter_RAP	0.442	0.108	**0.554**	0.038
Jitter_Jitt	0.485	0.160	**0.530**	0.097

RSP PP intervals	Self to RBR	Base to RBR	Self to 5 BrPM	Base to 5 BrPM
N measures	0	0	0	0
Median *rho*	**0.105**	0.080	0.064	0.059
Q3 *rho*	**0.233**	0.153	0.208	0.166
Q1 *rho*	**0.037**	-0.034	-0.123	-0.021
**Top 4 measures**				
Jitter_Jitta	**0.506**	0.278	0.275	0.187
Jitter_Jitt	**0.500**	0.221	0.270	0.187
FD_S	0.475	0.139	**0.480**	0.167
FD_Amp / FD_M	0.450	0.153	**0.470**	0.181

Table S10. EDA data (detrended and deduplicated). Number of measures with *rho* > 0.7 and the descriptive statistics for these (Q1, median, Q3), followed by the values of the four measures with greatest maximum values (highest values in each row indicated by bold type). For no measures was *rho* > 0.7 for all four comparisons.

EDA	Self to RBR	Base to RBR	Self to 5 BrPM	Base to 5 BrPM
N measures	5	1	27	30
Median *rho*	0.490	0.374	**0.507**	0.418

Q3 *rho*	0.593	0.528	**0.734**	0.732
Q1 *rho*	0.258	0.158	**0.299**	0.254
**Top 4 measures**	(For no measure was *rho* > 0.7 for all 4 comparisons)			
Jitter_Jitta	0.682	0.674	**0.853**	0.832
FD_K	0.653	0.623	**0.816**	0.782
RMSSD	0.648	0.620	**0.814**	0.780
EPP SD1_1	0.643	0.616	**0.814**	0.776

Correlation analysis thus indicates that agreement for the RRi and EDA measures was *less* between Self-paced and RBR breathing than between the other paired trials analysed, but that agreement between *some* of the raw Respiration and RSP interval measures was indeed greater for the Self-paced/RBR pair than for the other trial pairs.

However, counts of pairs with numbers of measures having *rho* > 0.7 did not support the hypothesis that self-paced breathing predicts RBR, for the EDA data particularly.

SM5.2. Correlations within 'families' of measures, and between individual measures when applied to different data types (RRi, respiration and EDA)

SM5.2.1. Correlations between fractal dimensions within the same dataset

Twenty-three FD measures were calculated for the RRi (4R) data, including the DynamicalSystems.jl version of FD_H, with 22 for the RRi (noR and 10R), RSP and EDA data, and 14 for the respiration interval data (which were too short for calculation of the eight NLD variants). For all eight data types taken together, there were 2923 positive and 508 negative correlations with |*rho*| > 0.7.

As already noted, resampling of the RRI data affected results of the Conover test. Here we observed that resampling also affected how many correlations occurred with Spearman's *rho* > 0.7 (or < -0.7). For all three data types (noR, 4R and 10R), more positive correlations occurred during self-paced than externally paced breathing (Figure SM3), and fewer negative correlations (which appeared to increase in number at higher breathing rates, particularly for the 10R RRi data).

For the respiration interval data, again more positive correlations occurred during self-paced than externally paced breathing (Figure SM3(b)), but this pattern was not observed in the raw RSP or EDA data (Figure SM3(c)).

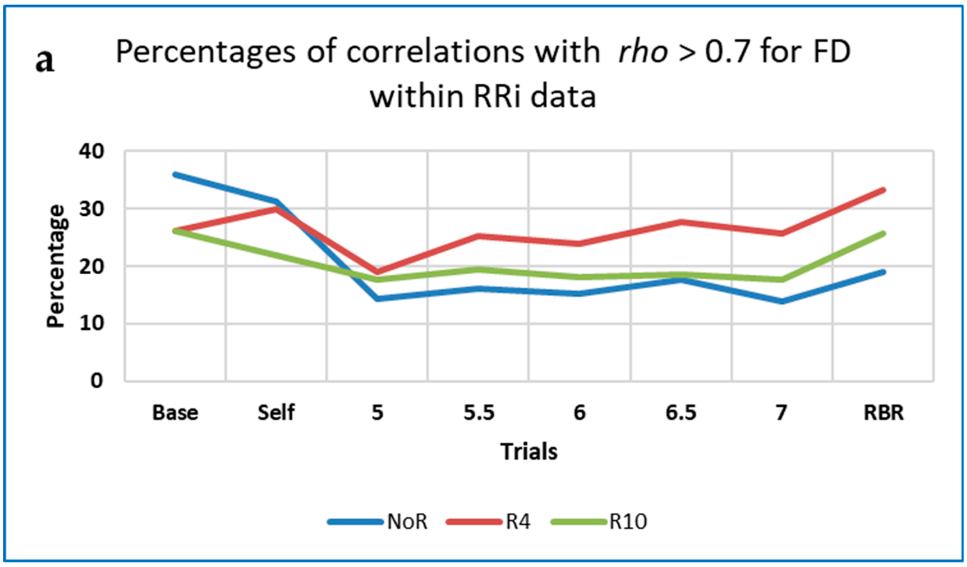
a
Percentages of correlations with rho > 0.7 for FD within RRi data
Percentage
40
30
20
10
0
Base
Self
5
5.5
6
6.5
7
RBR
Trials
NoR
R4
R10

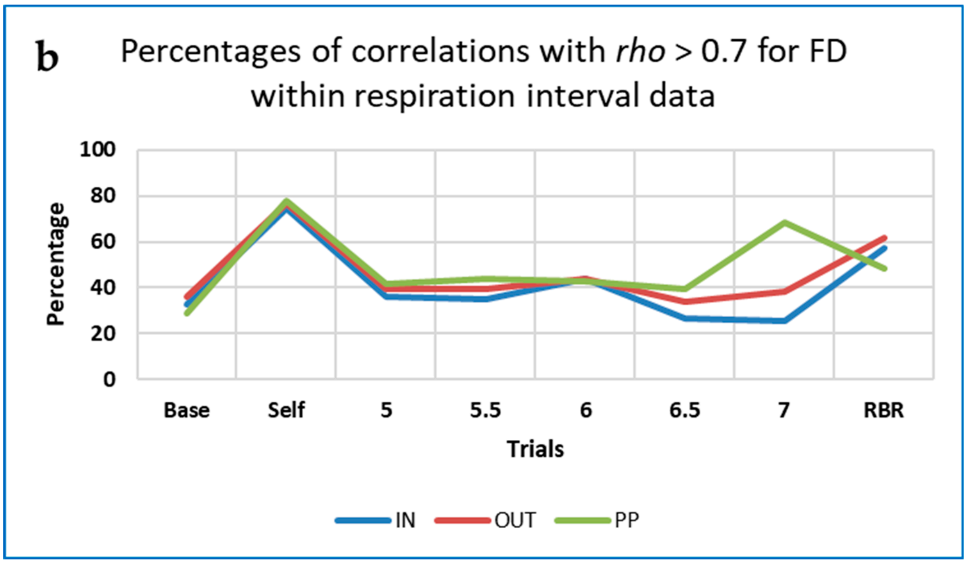
b
Percentages of correlations with rho > 0.7 for FD within respiration interval data
Percentage
100
80
60
40
20
0
Base
Self
5
5.5
6
6.5
7
RBR
Trials
IN
OUT
PP

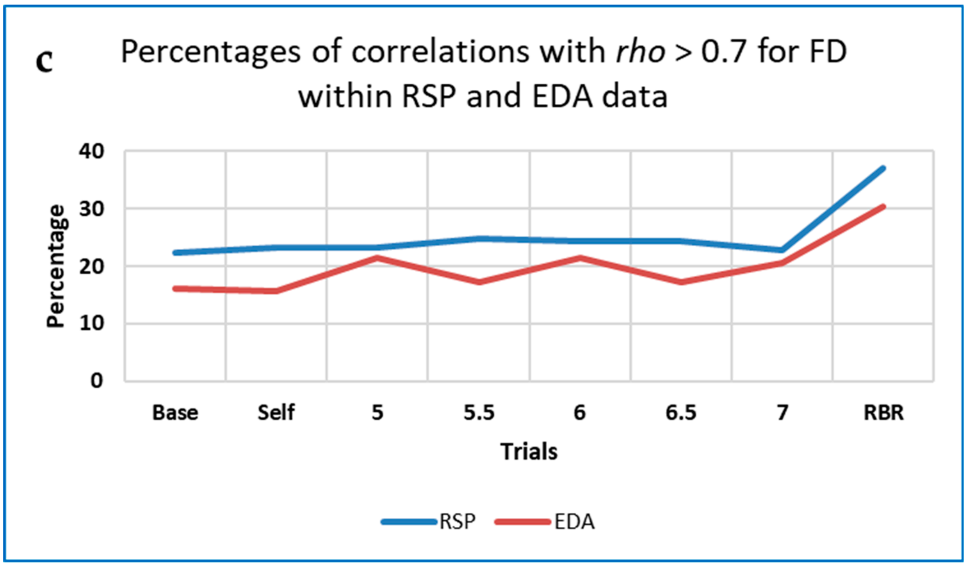
c
Percentages of correlations with rho > 0.7 for FD within RSP and EDA data
Percentage
40
30
20
10
0
Base
Self
5
5.5
6
6.5
7
RBR
Trials
RSP
EDA

Figure S3. Percentages of correlations between FD measures with Spearman's *rho* > 0.7, *within* the different data types: (a) RR interval data; (b) Respiration interval data (IN, OUT or PP); (c) Raw respiration (RSP) and Electrodermal activity (EDA) data. Note that y-axes are at different scales.

SM5.2.2. Correlations between datasets for individual fractal dimension measures

Interestingly, for the correlations *between* (rather than within) the different data types with *rho* > 0.7, a similar pattern of more positive correlations during self-paced than externally paced breathing was found for nine of the CEPS FD measures. It did not occur for the eight NLD variants, FD_K, FD_M or FD_S, FD_Amp or FD_Sign (and only partially for the box-counting algorithm of Meerwijk and van der Linden) [20] (Figure SM4).

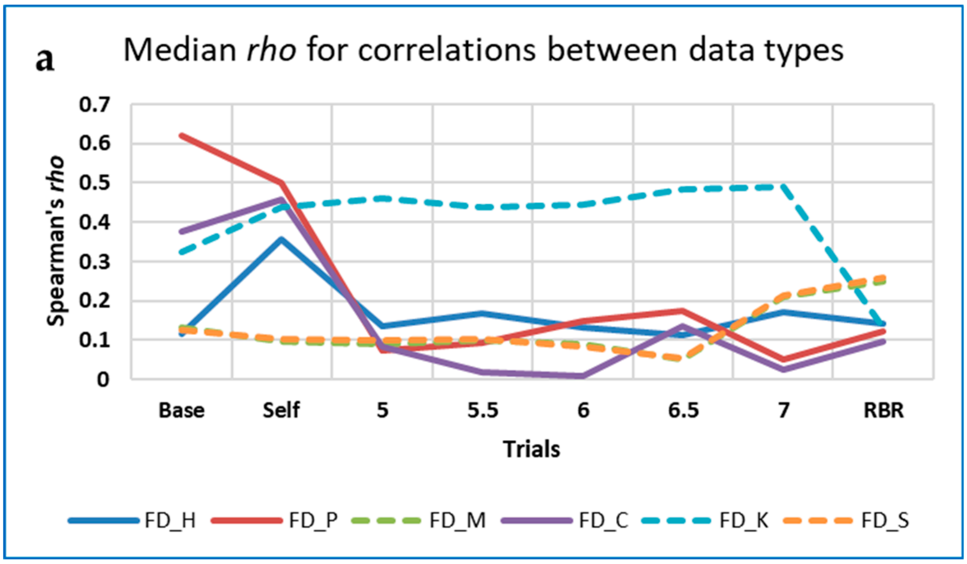

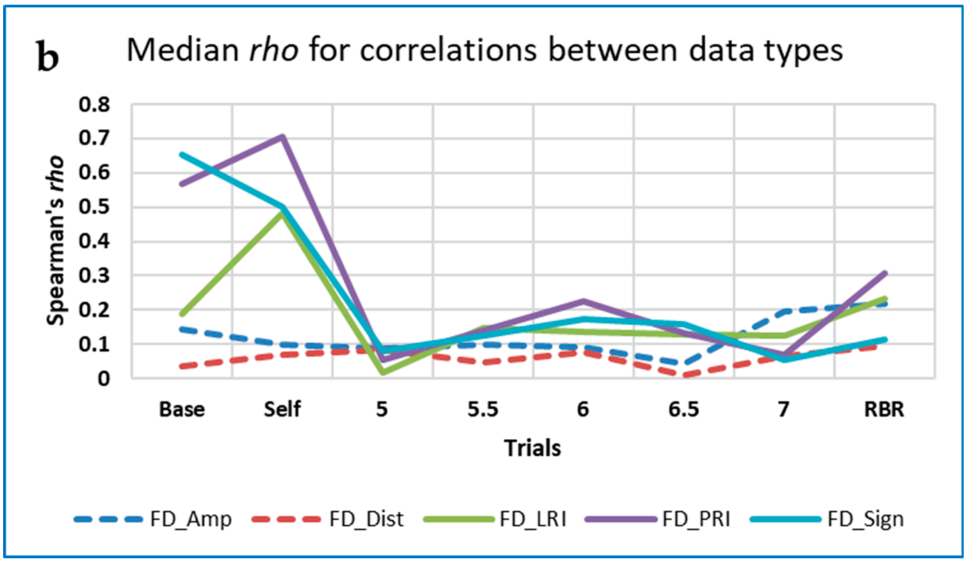

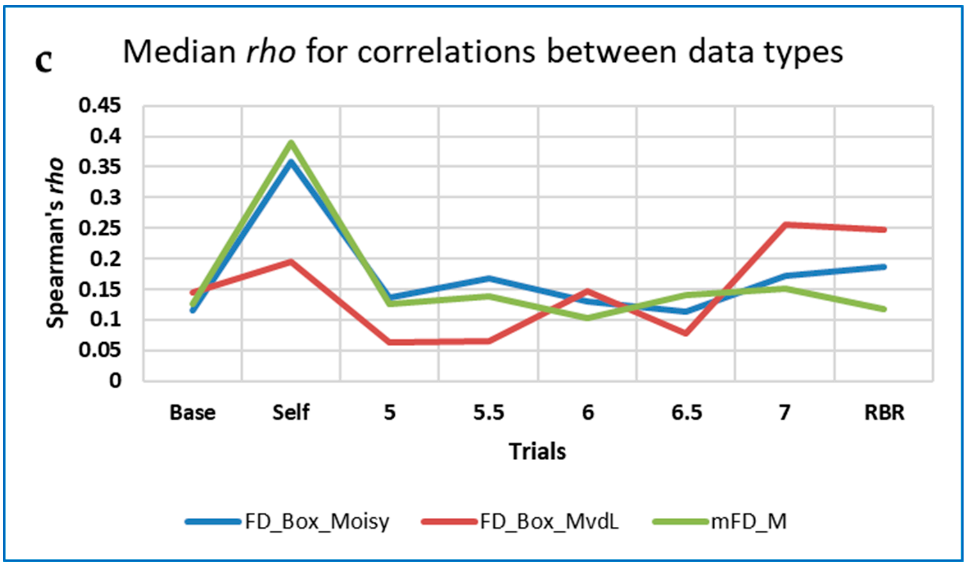

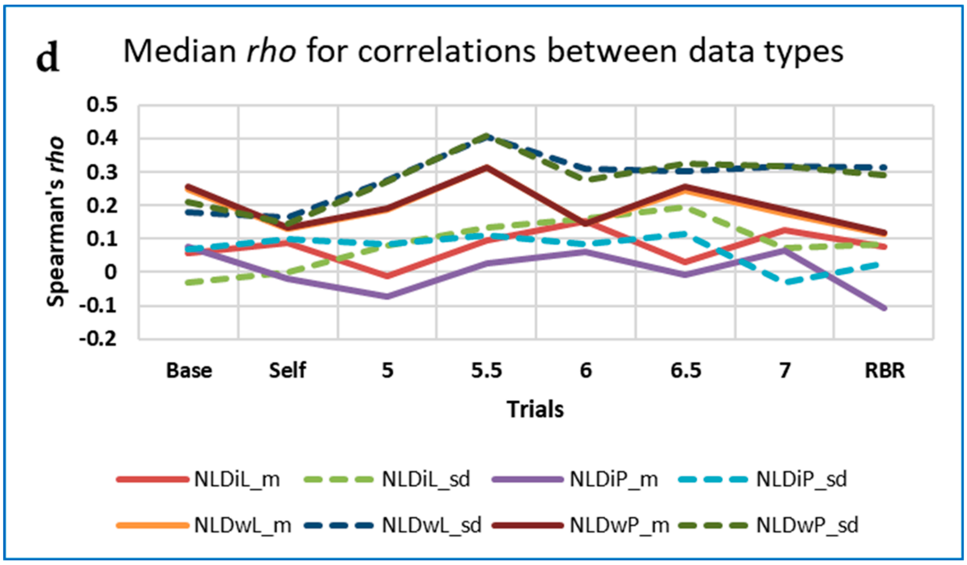

Figure S4. Median correlations (Spearman's *rho*), for FD measures *between* the different data types: (a) Methods named after their originators; (b) Methods of Kizlaitienė and Tamulevičius [21]; (c) Box-count and related methods [19,20,22,23]; (d) Kalauzi's NLD variants [8]. Note that y-axes are all at different scales. (For references, see the main paper.)

SM5.2.3. Correlations between HRA indices within the same dataset

Analysis was restricted to the five 'classical' HRA measures and 11 derived from Poincaré plots, as above. As for the FD measures, resampling of the RRI data affected how many correlations occurred with Spearman's *rho* > 0.7 (or < -0.7). For all three data types (noR, 4R and 10R), *fewer* correlations – whether positive or negative – occurred during self-paced than externally paced slow breathing (Figure SM5(a)). Note that, in a general sense, patterns of changes with respiration rate were similar for the noR, 4R and 10R data.

For the respiration interval data, again *fewer* positive correlations occurred during self-paced than externally paced breathing (Figure SM5(b)), but this pattern was not observed in the raw RSP or EDA data (Figure SM5(c)).

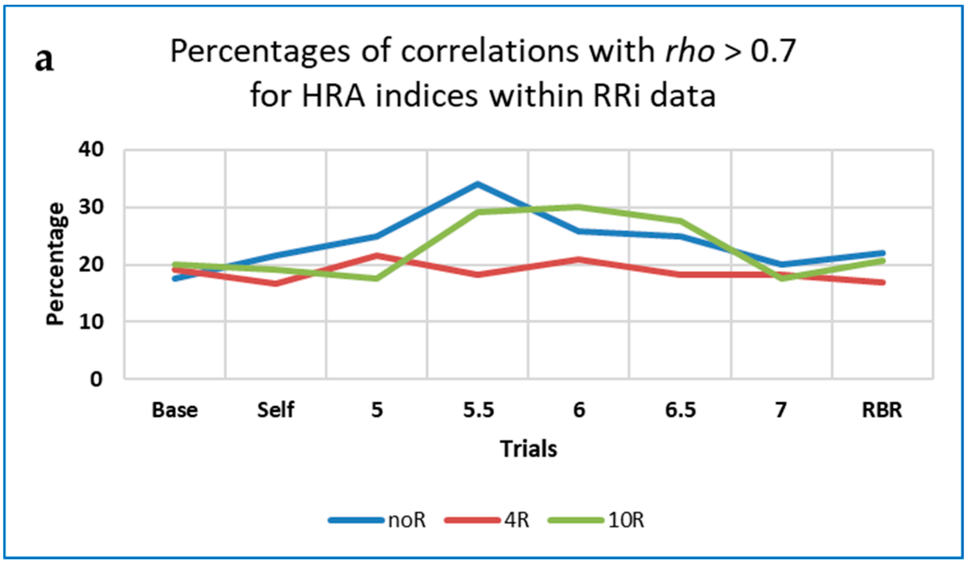
a
Percentages of correlations with rho > 0.7
for HRA indices within RRi data
40
30
20
10
0
Percentage
Base
Self
5
5.5
6
6.5
7
RBR
Trials
noR
4R
10R

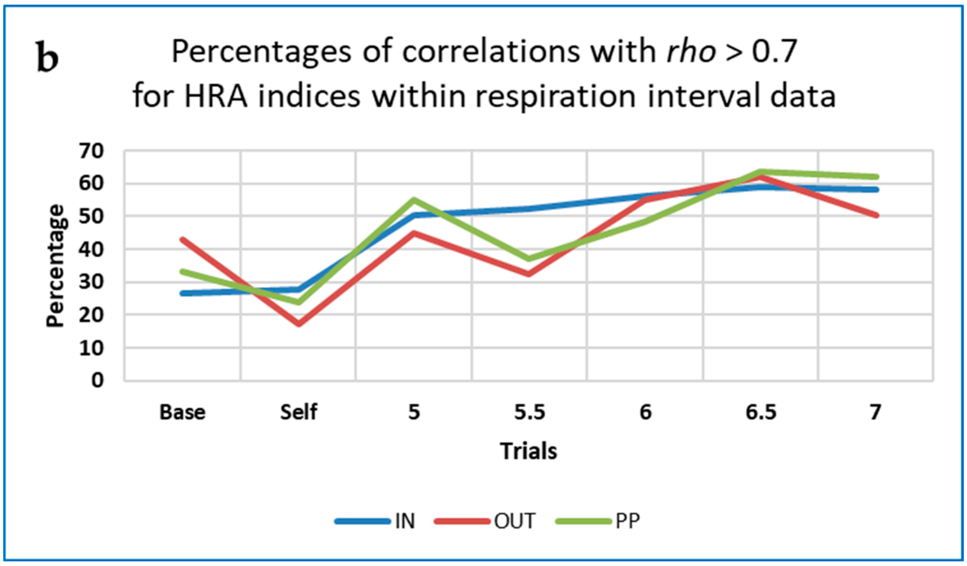
b
Percentages of correlations with rho > 0.7
for HRA indices within respiration interval data
70
60
50
40
30
20
10
0
Percentage
Base
Self
5
5.5
6
6.5
7
Trials
IN
OUT
PP

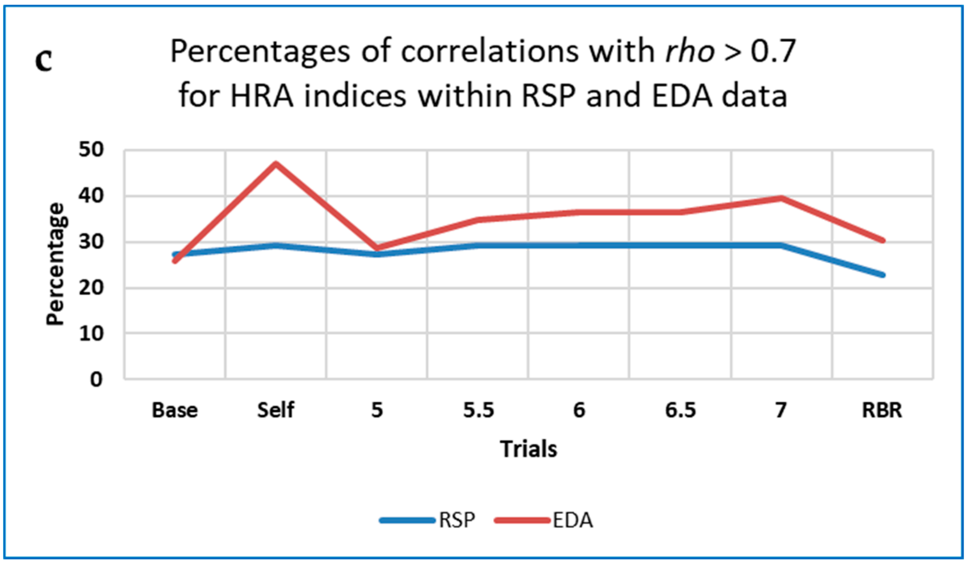
c
Percentages of correlations with rho > 0.7
for HRA indices within RSP and EDA data
50
40
30
20
10
0
Percentage
Base
Self
5
5.5
6
6.5
7
RBR
Trials
RSP
EDA

Figure S5. Percentages of correlations between HRA measures with Spearman's *rho* > 0.7, for the different data types: (a) RR interval data; (b) Respiration interval data (IN, OUT or PP); (c) Raw respiration (RSP) and Electrodermal activity (EDA) data. Note that y-axes are all at different scales.

SM5.2.4. Correlations between datasets for individual HRA indices

Correlations *between* (rather than within) the different data types with *rho* > 0.7 occurred for the five classical indices, Guzik's subsidiary descriptors and the normalised HRA measures. Strong correlations were not found between RRi and respiration interval data, nor between either of these and the raw RSP or EDA data. The respiration interval data was too short to allow meaningful calculation of Rohila's ASI; again, correlations were not calculated for the composite RBR trial.

For the RRi data, normalised measures $C1_a$ and $C1_d$ behaved differently from normalised measures ($C2_a$ and $C2_d$, as shown in Figure SM6(a), whereas the subsidiary descriptors for the respiration interval data differed according to whether they were 'up' or 'down' (Figure SM6(c)). For the RRi data, on the other hand, although there were differences between the 'up' and 'down' subsidiary descriptors, they showed relatively little variance with respiration rate (Figure SM6(b)). For this reason, $SD1_{up}$ and $SD1_{down}$ are not shown. The five classical HRA indices for the RRi data are shown in Figure SM6(d), with stronger correlations for PI and GI than the other three, and for the RSP interval data in Figure SM6(e) with poor correlations for PI between the IN, OUT and PP data.

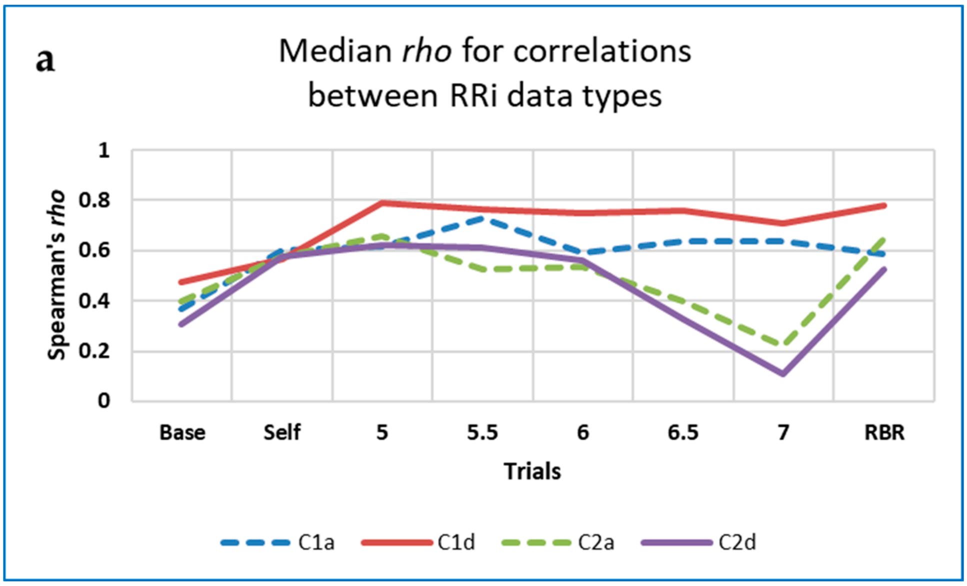

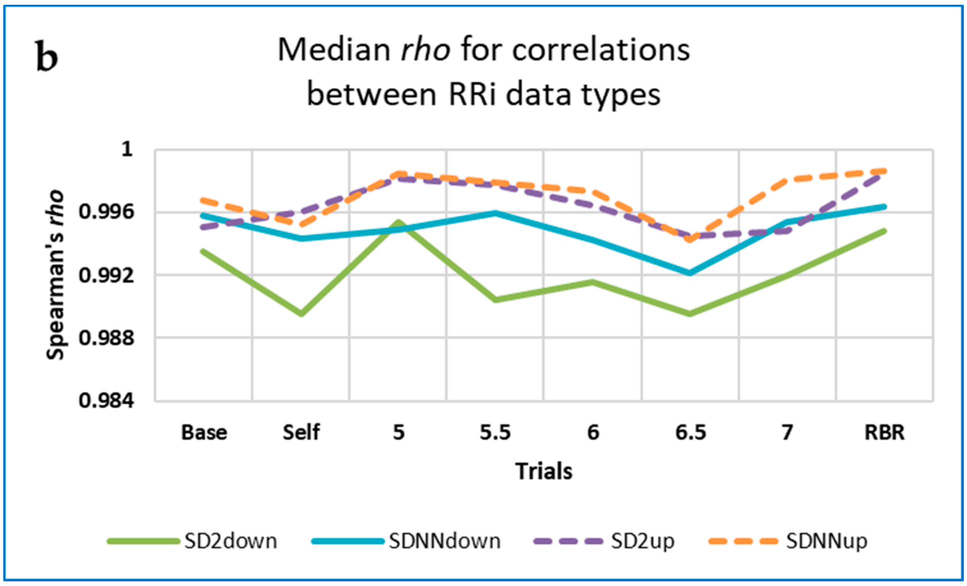
b
Median rho for correlations between RRi data types
Spearman's rho
1
0.996
0.992
0.988
0.984
Base
Self
5
5.5
6
6.5
7
RBR
Trials
SD2down
SDNNdown
SD2up
SDNNup

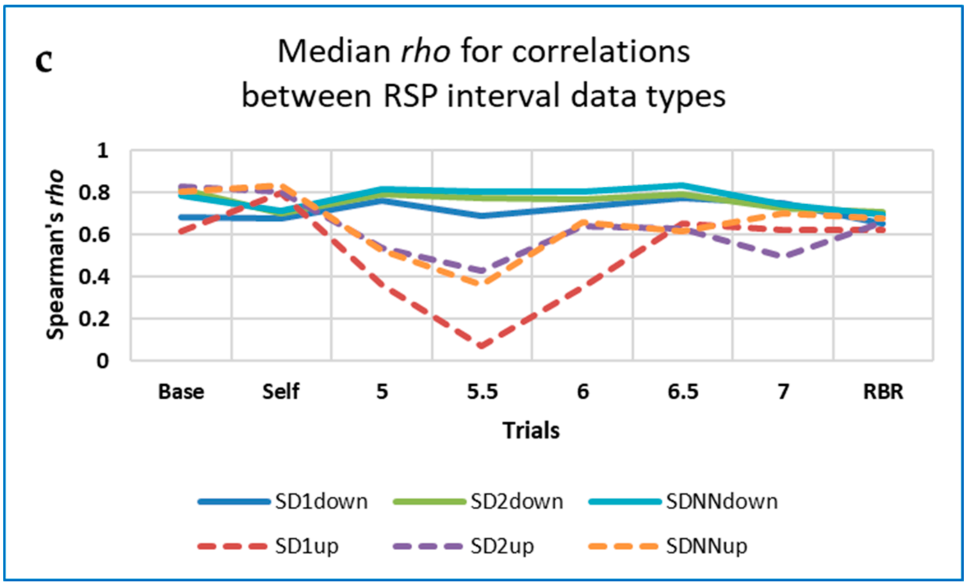
c
Median rho for correlations between RSP interval data types
Spearman's rho
1
0.8
0.6
0.4
0.2
0
Base
Self
5
5.5
6
6.5
7
RBR
Trials
SD1down
SD2down
SDNNdown
SD1up
SD2up
SDNNup

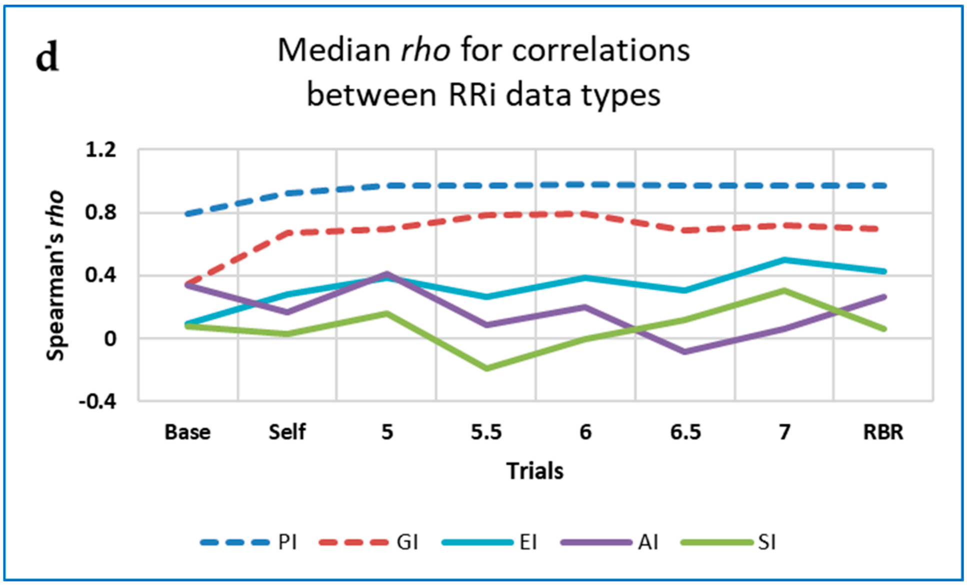
d
Median rho for correlations between RRi data types
Spearman's rho
1.2
0.8
0.4
0
-0.4
Base
Self
5
5.5
6
6.5
7
RBR
Trials
PI
GI
EI
AI
SI

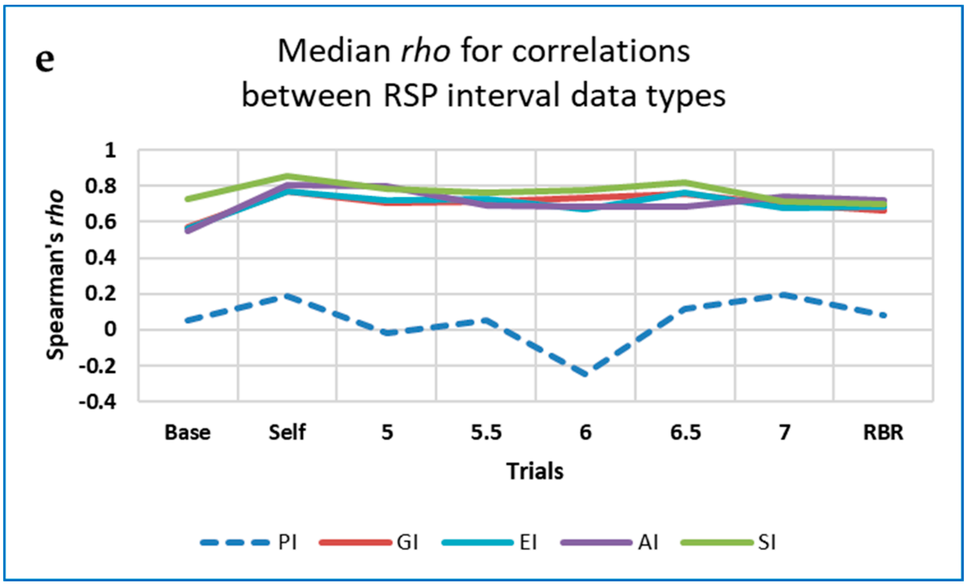

Figure S6. Median correlations between HRA measures with Spearman's *rho* > 0.7, *between* the different data types: (a) Normalised measures for RRi data types; (b) Subsidiary descriptors for RRi data types; (c) Subsidiary descriptors for RSP interval data types; (d) Classical HRA indices for RRi data types; (e) Classical HRA indices for RSP interval data types. Note that y-axes are not all to the same scale.

SM5.2.5. Correlations between the PE-based measures within the same RRi or respiration interval dataset

Eight PE-based measures were included in this analysis (mPE1, EPE, ImPE, mPM_E, RPE, TPE, CPEI and PJSC), but not AAPE, for which the respiration interval data were too short. Raw RSP and EDA data were also not analysed. Figure SM7 shows the results for the seven trials.

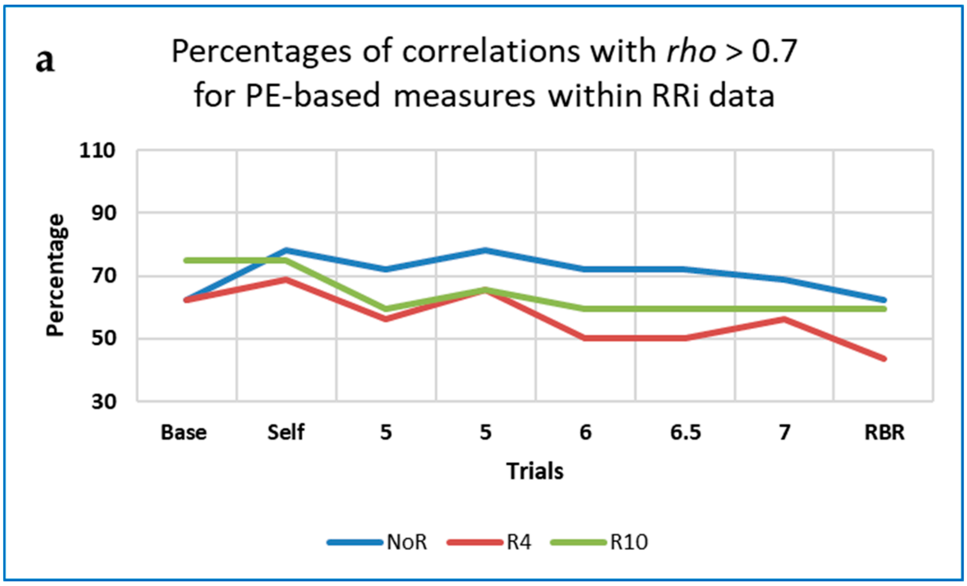

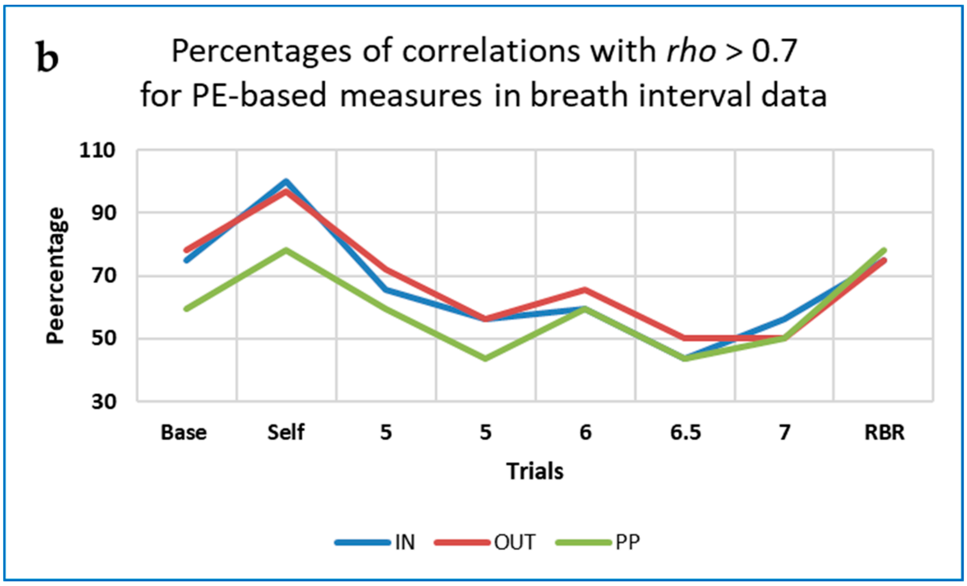

Figure S7. Percentages of correlations between PE-based measures with Spearman's *rho* > 0.7, for the different data types: (a) RR interval data; (b) Respiration interval data (IN, OUT or PP).

Correlations for the equally resampled RRi data occurred more frequently during Self-paced breathing than during externally paced breathing, but this was not the case for the non-resampled RRi data. Correlations were also marked during Self-paced (but not externally paced) breathing for the PE-based measures derived from the respiration (breath) interval data, occurring for all 32 possible correlations for the INbreath data.

SM5.2.6. Correlations between data types for eight individual PE-based measures

Correlations for these eight measures across six data types (three RRi, three respiration interval) are shown in Figure SM8. Although PJSC is quite different from the other seven measures, all show the same pattern of greater correlation during Self-paced breathing, with correlation minimal when breathing at 5.5 BrPM.

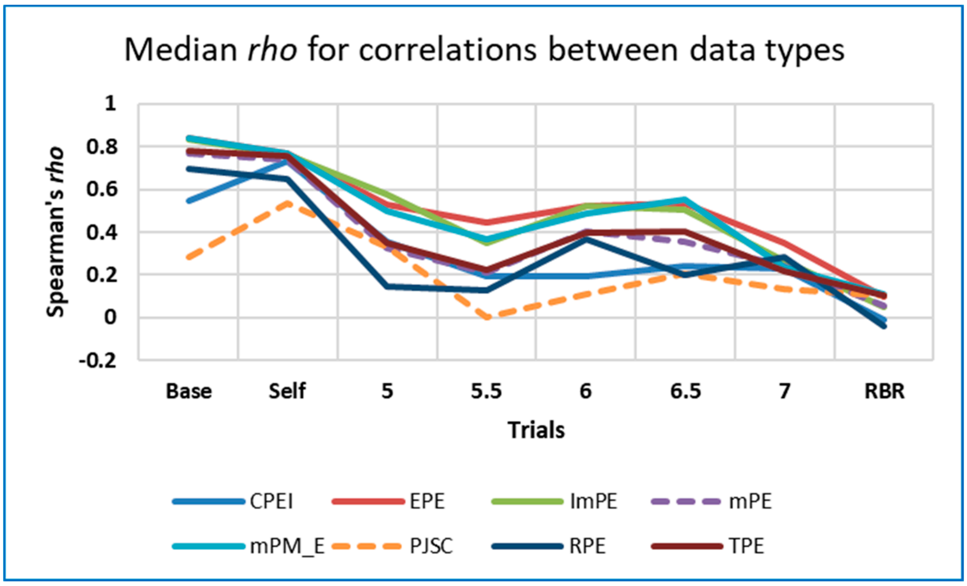

Figure S8. Median correlations between PE-based measures with Spearman's *rho* > 0.7, *between* six different data types: RRi noR, 4R and 10R, and IN, OUT and PP respiration intervals.

Comparing the results in Figures SM3 to SM8, it is clear that correlations with *rho* > 0.7 within the different datasets occur more frequently for the PE-based family of measures, and that median values of correlations between the same measure in the different datasets are also strongest for these measures.

In summary, correlations of measures within or between data types would thus appear to offer fertile ground for further investigation.
